# Axonal-Transport-Mediated Gene Transduction in the Interior of Rat Bone

**DOI:** 10.1371/journal.pone.0013034

**Published:** 2010-09-28

**Authors:** Toshitaka Okabayashi, Kuniaki Nakanishi, Toyokazu Tsuchihara, Hiroshi Arino, Yasuo Yoshihara, Susumu Tominaga, Maki Uenoyama, Shinya Suzuki, Masataka Asagiri, Koichi Nemoto

**Affiliations:** 1 Department of Orthopaedic Surgery, National Defense Medical College, Saitama, Japan; 2 Department of Laboratory Medicine, National Defense Medical College Hospital, Saitama, Japan; 3 Department of Pathology and Laboratory Medicine, National Defense Medical College, Saitama, Japan; 4 Division of Environmental Medicine, National Defense Medical College Research Institute, Saitama, Japan; 5 Department of Chemistry and Biochemistry, University of California San Diego, San Diego, California, United States of America; University of Florida, United States of America

## Abstract

**Background:**

Gene transduction has been considered advantageous for the sustained delivery of proteins to specific target tissues. However, in the case of hard tissues, such as bone, local gene delivery remains problematic owing to anatomical accessibility limitations of the target sites.

**Methodology/Principal Findings:**

Here, we evaluated the feasibility of exogenous gene transduction in the interior of bone via axonal transport following intramuscular administration of a nonviral vector. A high expression level of the transduced gene was achieved in the tibia ipsilateral to the injected tibialis anterior muscle, as well as in the ipsilateral sciatic nerve and dorsal root ganglia. In sciatic transection rats, the gene expression level was significantly lowered in bone.

**Conclusions/Significance:**

These results suggest that axonal transport is critical for gene transduction. Our study may provide a basis for developing therapeutic methods for efficient gene delivery into hard tissues.

## Introduction

Defective bone healing is a major clinical problem. The ability to promote osteogenesis in a controlled way would be beneficial for the treatment of bone fractures and large segmental bone defects, for the fixation of artificial joints and for avoiding nonunion or delayed union of fractures [1]. When therapeutic genes, such as bone morphogenetic protein (BMP) cDNAs, are supplied to bone fractures and bone defects, they can offer sustained delivery of proteins to a local area. Thus, local gene delivery into bone has a huge therapeutic potential for the treatment of bone diseases. *Ex vivo* and *in vivo* therapeutic procedures for gene delivery to local bone tissue have been reported {reviewed in [2]}: i) the gene is introduced into cultured cells, which are then implanted into the patient (*ex vivo* transfer) [3]–[6], or ii) the gene is transferred directly into the target sites (*in vivo* transfer) [7]–[11]. *Ex vivo* methods are safer in terms of the fact that the toxic or immunogenic effects of reagents are minimized; however, they pose risks of potential bacterial and/or infectious contamination caused by manipulating cells *ex vivo*, and of mix-ups of samples and the phenotypic transformation of the cells. On the other hand, *in vivo* gene therapy is technically simple and less invasive [12]. In the case of calcified tissues, however, the *in vivo* local delivery of an exogenous gene remains problematic because of the limited accessibility into the bone tissue [13]. To avoid the possible defects caused by *ex vivo* and *in vivo* gene transfer, systemic gene transfer via the vascular system has been tested for the reversal of osteopenia in ovariectomized mice [14], [15]. However, systemic gene delivery has been reported to cause severe side effects including strong immune responses to reagents related to the gene delivery [9], [16], [17].

Anatomically, sensory and sympathetic nerve fibers are present in bone, and they form dense parallel networks around blood vessels contiguous to bone trabeculae in close contact with bone cells [18], and play an important role in bone metabolism [19]–[22]. Thus, close anatomical and functional relationships have been found between bone and the nervous system. Previously, we demonstrated that a nonviral vector could transfer a gene successfully into peripheral nerves, dorsal root ganglia, and the spinal cord via retrograde axonal transport from an injected muscle [23]. In this study, to test the hypothesis that genes transfer to bone tissue via complicated networks between bone and the nervous system, we evaluated the efficacy of gene expression in the interior of bone via axonal transport following intramuscular injection of a nonviral vector [HVJ (hemagglutinating virus of Japan) envelope].

## Materials and Methods

### Ethics Statements

This experimental study was carried out in strict accordance with the recommendations in the Guide for the Care and Use of Laboratory Animals of the National Institutes of Health. The protocol was approved by the Committee on the Ethics of Animal Experiments of the National Defense Medical College.

### Construction of luciferase plasmid DNA

To evaluate transfection efficiency, we used a pcDNA/GL3 luciferase plasmid vector, which was a kind gift from Dr. Yasuhumi Kaneda (Osaka University, Japan). The pcDNA/GL3 luciferase plasmid vector (7.4 kb) was constructed by cloning the luciferase gene from the pGL3-Promoter Vector (Promega, WI, USA) into pcDNA3 (5.4 kb) (Invitrogen, CA, USA) at the *Hin*d III and *Bam*H I sites. The pGL3 promoter vector containing the SV40 promoter was inserted into the pcDNA3 vector containing a cytomegalovirus promoter (5.4 kbp) between the *Hin*d III and *Bam*H I sites.

### Preparation of an HVJ envelope/luciferase gene complex vector

The HVJ envelope/luciferase gene complex vector was prepared using an HVJ envelope vector kit (GenomOne-Neo; Ishihara Sangyo, Osaka, Japan). Briefly, freeze-dried HVJ envelopes (100 µl) were reconstituted and placed into a micro-test tube. An incorporation reagent (10 µl) was added to the HVJ envelope suspension and agitated. The mixture was centrifuged (12000 rpm, 4°C, 10 min), and the supernatant was discarded. The sediment was suspended in the buffer (25 µl) supplied with the kit and mixed with luciferase plasmid DNA/TE solution (25 µl), to yield a vector concentration of 4 µg/µl. After being left to stand for 5 min, the HVJ envelope/luciferase gene complex vector (1 µg/µl) was used immediately.

### Injection of an HVJ envelope/luciferase gene complex vector into the tibialis anterior

Eighty-eight male Wistar rats, approximately 7 weeks old and weighing 150–170 g, were used in this study. Seventy-two rats were used for the evaluation of gene expression [luciferase activity and reverse transcription polymerase chain reaction (RT-PCR)], nine were used to assess gene distribution, and the remaining seven were subjected to sciatic nerve transection. They were housed in a temperature-controlled room with a 12-hour light-dark cycle.

All surgical procedures were carried out under sodium pentobarbital anesthesia (30–50 mg/kg body weight, intraperitoneally injected). HVJ envelopes (100 µl) containing 100 µg of luciferase reporter plasmid were carefully injected percutaneously into the proximal one-third of the tibialis anterior muscle of the right hindlimb via a 27-gauge needle (Terumo, Atsugi, Japan), each injection taking 3 to 5 min so as to avoid leakage out of the fascia.

### Analysis of luciferase activity

**Figure 1:**
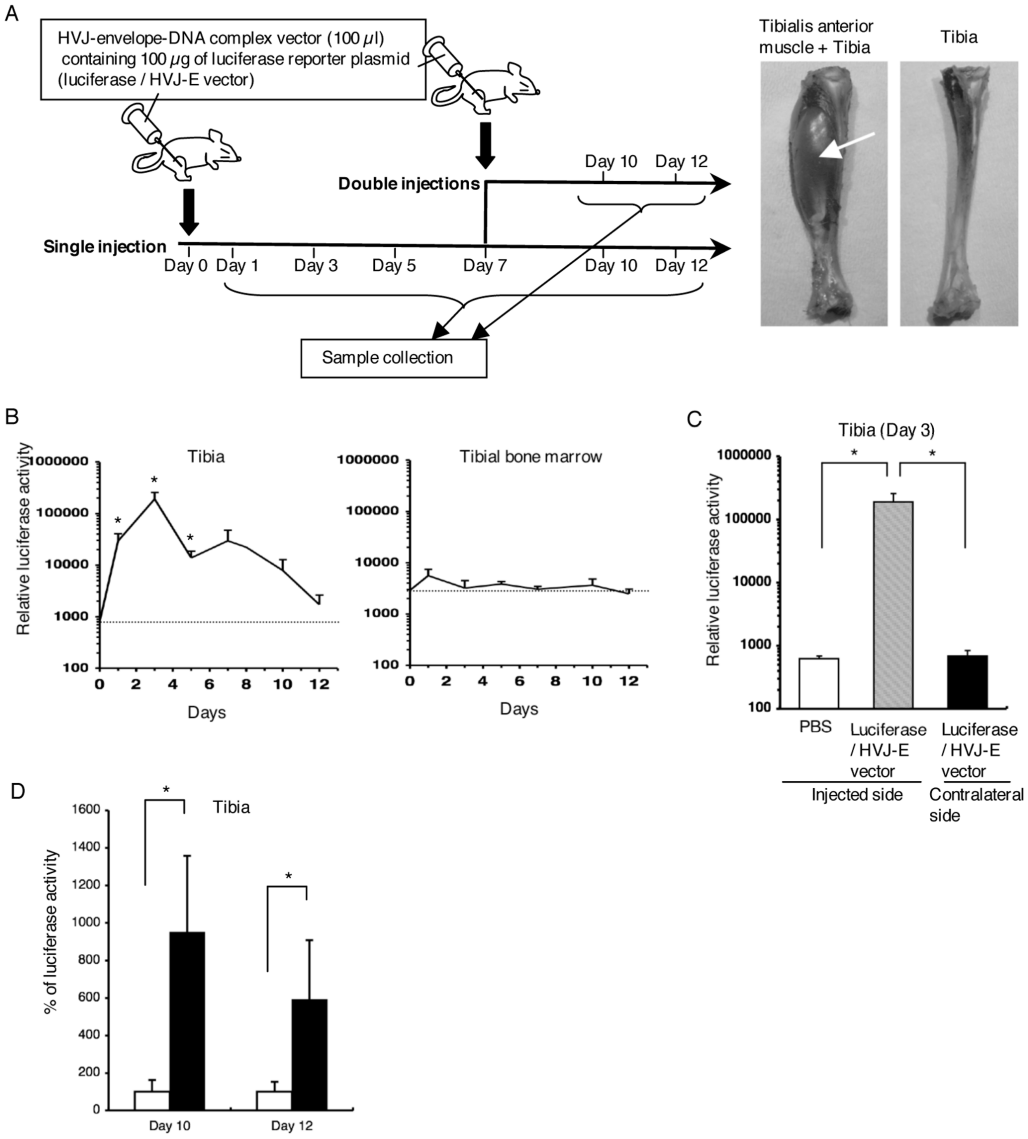
Gene expression in the interior of the bone by intramuscular administration of an HVJ-envelope-DNA complex. (A) Experimental design of gene transfer. A luciferase/HVJ-E vector (100 µl) containing 100 µg luciferase plasmid DNA was carefully injected percutaneously into the proximal one-third of the tibialis anterior muscle of the right hindlimb. The first gene administration was on day 0, and the second on day 7. Samples were harvested on days 1, 3, 5, 7, 10, and 12 after the first administration of a luciferase/HVJ-E vector (each group: n = 7). Luciferase activity was measured using a luciferase assay system. Pictures show tibia with tibialis anterior muscle (arrow) and tibia from which the periosteum was thoroughly stripped to avoid any contamination with muscles. (B) Relative luciferase activity (RLU/mg protein) in the ipsilateral tibia (without periosteum), and bone marrow of the ipsilateral tibia. Error bars: SEM. Each group: n = 7. The dotted lines indicate the average levels of control samples, which were harvested at each time point after PBS injection (n = 6). Error bars: SEM. *p<0.05. (C) Relative luciferase activity (RLU/mg protein) in the tibia (without periosteum) on day 3 after gene transfer. Black bar: the activity in the contralateral tibia (n = 7), gray bar: the activity in the ipsilateral tibia (n = 7), white bar: the activity in the tibia after injection of PBS as a control (n = 6). Error bars: SEM. *p<0.05. (D) Comparison of luciferase activities of the ipsilateral tibia between single and repeated gene transfers on days 10 and 12. When luciferase activity in the ipsilateral tibia without a second gene transfer is considered as 100%, its activity in the ipsilateral tibia with second gene transfer on day 7 is shown as a percentage. Black bars: rats with the second gene transfer on day 7, white bars: rats without a second gene transfer. Error bars: SEM. Each group: n = 7. *p<0.05.

Luciferase activity was measured using a luciferase assay system (Promega, WI, USA). Forty-two rats were used to evaluate the transfection efficiency of an HVJ-envelope complex vector. The injected muscle (tibialis anterior), bilateral sciatic nerves, bilateral dorsal root ganglia (L4, L5), bilateral femora, bilateral tibiae, and bone marrows from bilateral femora and tibiae were harvested as specimens on days 1, 3, 5, 7, 10, and 12 after the transfer of a pcDNA/GL3 luciferase plasmid (n = 7 for each day after transfection) (Fig. 1A). In addition, fourteen rats were used to evaluate the transfection efficiency of an HVJ-envelope complex vector following a second gene transfer (performed on day 7 after the first gene transfer, using the same injection procedure into the same place in the muscle as the first). This time, specimens were harvested on days 3 and 5 after the second gene transfer (n = 7 for each day after the second transfection) (Fig. 1A). Twelve rats were used as controls. Samples were harvested on days 1 and 3 after the injection of phosphate buffered saline (PBS) (100 µl) (n = 6 for each day after injection). Bone marrows were flushed out, and periostea were thoroughly stripped from bones before sampling to avoid contamination with muscles (Fig. 1A). Then, the samples were rapidly frozen in liquid nitrogen and homogenized in a cell-culture lysis reagent [in advance, mineral bones were crushed using a Cryo-Press (Microtec, Chiba, Japan)]. Subsequently, the tissue lysates were briefly centrifuged (12000 rpm, 4°C, 3 min), and 20 µl of supernatant was mixed with 100 µl of luciferase assay reagent. The luminescence reaction was quantified using a Lumat LB 9507 (Berthold Technologies, Bad Wildbad, Germany), and the residual supernatant was used in a colorimetric assay of protein concentration, for which a DC protein assay system (Bio-Rad Laboratories, CA, USA) and a Model 680 Microplate Reader (Bio-Rad Laboratories, CA, USA) were used. The luminescence reaction, as adjusted by the concentration of protein, was used as an index of luciferase activity.

### Immunohistochemistry for luciferase and calcitonin gene-related peptide (CGRP)

Nine rats were used for immunohistochemical staining. Tibialis anterior muscles, sciatic nerves, dorsal root ganglia, and tibiae were harvested on day 3 after the first gene transfer. The specimens (except for bones) were then rapidly embedded in an optimal cutting temperature (OCT) compound and frozen at −80°C. Sagittal sections of 6 µm thickness were cut serially on a cryostat and mounted on silane-coated slides. Specimens of bones were embedded in carboxymethyl cellulose (CMC) compound and frozen at −80°C. Tibial cross sections of 10 µm thickness [cut using a tungsten carbide blade after trimmed surfaces had been covered with Cryofilm (Finetec, Tokyo, Japan)] were mounted on slides. After being air-dried for 30 min, the sections were immersed and fixed in 0.1 M phosphate buffer (pH 7.4) containing 4% paraformaldehyde, and rinsed in running water. Then, they were treated with 2% bovine serum albumin in PBS for 10 min at room temperature to block nonspecific protein binding, and incubated overnight at 4°C in a humidified chamber with a polyclonal antibody against recombinant firefly luciferase (Promega, WI, USA) diluted at 1∶100. The sections were rinsed in PBS and incubated with an anti-goat IgG FITC-conjugated secondary antibody (Chemicon, CA, USA) diluted at 1∶50 for 2 h. After further rinsing in PBS, the sections were finally immersed in 4′, 6-diamidino-2-phenylindole (DAPI) for 15 min to stain nuclei.

To identify the location of intra-osseous sensory nerve fibers, tibial serial sections were stained with an anti-calcitonin gene-related peptide (CGRP) antibody (Biomol, PA, USA). The sections were air-dried for 10 min at room temperature and fixed in 0.1 M phosphate buffer (pH 7.4) containing 4% paraformaldehyde, and rinsed in running water. Then, they were treated with 3% H_2_O_2_ in 90% methanol for 5 min to inactivate endogenous peroxidase. They were rinsed with PBS for 30 min and treated with 2% bovine serum albumin in PBS for 10 min at room temperature to block nonspecific protein binding. Then, they were stained with a rabbit anti-CGRP polyclonal antibody (Biomol, PA, USA) diluted at 1∶500 for 3 days at 4°C in a humidified chamber. The sections were rinsed in PBS and incubated for 24 h in a Histofine simple stain MAX-PO (rabbit) secondary antibody (Nichirei, Tokyo, Japan) at 4 °C in a humidified chamber. After further rinsing in PBS, they were immersed in peroxidase with 3,3-diaminobenzidine (DAB) chromogen. Immunohistochemical stainings for luciferase and CGRP were performed more than 3 times for each specimen of nine rats.

### RT-PCR

Four rats were used for RT-PCR analysis. The expression of luciferase mRNA in the injected muscles (tibialis anterior), ipsilateral femora, ipsilateral tibiae, and bone marrows from ipsilateral femora and tibiae on day 3 after the first gene transfer was examined (n = 4). Periostea were entirely stripped from tibiae and femora and different forceps were used for each sample collection so that bone tissues would not be contaminated with attached muscles. Total RNA was isolated using ISOGEN (Nippon Gene, Tokyo, Japan) with ethanol precipitation. The pcDNA/GL3 luciferase plasmid vector cut with the restriction enzyme *Xho* I was used as the control. RT-PCR was performed using an amplification reagent kit (TaqMan EZ RT-PCR kit; Applied Biosystems, CA, USA) with a primer specific for firefly luciferase (product length, 261 bp). Glyceraldehyde 3-phosphate dehydrogenase (GAPDH) mRNA levels were used as internal controls. The primer was synthesized using an automated DNA synthesizer. Sequence information and the thermocycling conditions were as follows. luciferase primers: sense, 5′-ACTGCCTGCGTGAGATTCTC-3′; antisense, 5′-CAGAGTGC TTTTGGCGAAGA-3′; GAPDH primers: sense, 5′-CTTCACCACCATGGAGAAGGC-3′; antisense, 5′-GGCATGGACTGTGGTCATGAG-3′; annealing temperature, 60°C; cycles, 40. The PCR product was separated by electrophoresis in a 3% agarose gel, and stained with ethidium bromide. Quantification was performed using Image J software (National Institutes of Health, Bethesda, USA; http://rsb.info.nih.gov/ij/).

We also performed RT-PCR using each sample and a primer specific for myosin heavy chain (MHC) IIb (product length, 197 bp). MHC IIb is localized in fast-twitch muscle. Sequence information and the thermocycling conditions were as follows: sense, 5′-CTGAGGAACAATCCAACGTC-3′; antisense, 5′-TTGTGTGATTTCTTCTGTCACCT -3′; annealing temperature, 59°C; cycles, 25. The PCR product was separated by electrophoresis in a 3% agarose gel, and stained with ethidium bromide.

### Sciatic nerve transection experiment

Seven rats were used for the sciatic nerve transection experiment. In the sciatic nerve transection group (n = 7), the unilateral (right) sciatic nerves were transected one day before injecting an HVJ-envelope complex vector. HVJ-envelope-DNA complex vector injection was performed in the right tibialis anterior muscles, and then these muscles, the ipsilateral sciatic nerves, dorsal root ganglia (L4 and L5), and tibiae (without bone marrows and periostea) were harvested on day 3 after the injection. Then, luciferase assay was performed as described above.

### Statistical analysis

Data are expressed as mean vlues ± standard error of mean (SEM). Dunnet's test was used for luciferase activity between the gene transfection group and the control group. Unpaired Student's *t*-test was used to analyze the difference between the luciferase activities of tibia with single and repeated gene transfers, and between the intact sciatic nerve group and the sciatic nerve transection group. P values less than 0.05 were considered to indicate statistical significance.

## Results

### Gene expression in the interior of the bone by intramuscular administration of an HVJ-envelope-DNA complex

We utilized a combination of a luciferase reporter gene plasmid [24] and a nonviral HVJ envelope vector [25] [hereafter referred to as a “luciferase/HVJ-E vector” (see also Materials and Methods section)] to examine the efficacy of gene induction in bones via neuronal transport following the intramuscular injection of a vector. The tibialis anterior muscles, sciatic nerves, dorsal root ganglia (L4 and L5), femora, tibiae, and bone marrows from bilateral femora and tibiae were harvested on days 1, 3, 5, 7, 10, and 12 after the administration of a luciferase/HVJ-E vector (Fig. 1A). A marked increase in luciferase activity was observed in the injected muscle, the ipsilateral nerve, and dorsal root ganglia (L4 and L5), as in our previous report [23]. To our surprise, the ipsilateral tibia showed a high increase in luciferase activity, while the tibial bone marrow and the contralateral tibia showed no increase (Figs. 1B and C). Next, we investigated whether repeated gene transfer could lead to a sustained luciferase gene expression in the bones. The second gene transfer led to upregulated luciferase activity in the ipsilateral tibia (Fig. 1D), confirming the validity of repeated gene transfer in our method. These results showed the feasibility of inducing a sustained gene expression in bones following injections of an HVJ-envelope-DNA complex vector into the muscle.

### Immunohistochemical analysis of luciferase gene products in the bone

**Figure 2:**
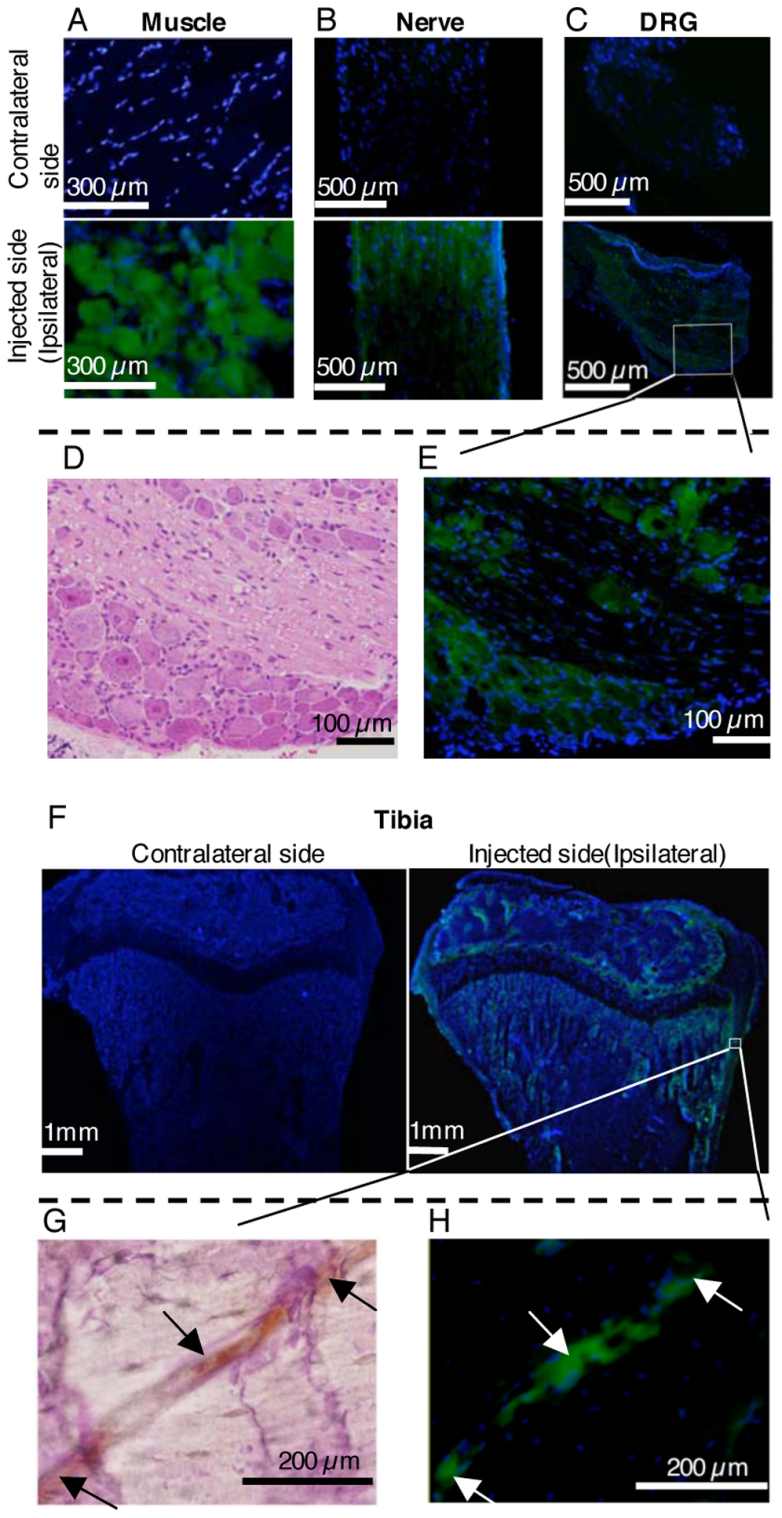
Immunohistochemical analysis of luciferase gene products (green) in the bone. Nuclei (blue) were stained with DAPI. (A) There were many immunoreactive muscle fibers in the injected muscle. (B) The ipsilateral sciatic nerve and (C) dorsal root ganglia were also immunoreactive. (D) Hematoxylin-eosin staining of different sections obtained from the same CMC compoud of (E). (E) Sensory neurons in the ipsilateral dorsal root ganglion showed luciferase protein expression. (F) The ipsilateral tibia showed immunoreactivity as well. (G) CGRP staining of the serial section of (H). CGRP protein was detected (arrows) in the area where luciferase protein was expressed in (H). (H) The luciferase protein was expressed (arrows) in a tube-like structure known as a Haversian or Volkmann's canal in the cortical bone. Immunohistochemical stainings were performed more than 3 times for each specimen and all the results were the same as shown here.

To further evaluate the detailed distribution of the introduced gene expression, immunohistochemical staining for luciferase was performed with an anti-luciferase antibody (more than 3 times for each specimen of nine rats). As shown in Figs. 2A to E, luciferase protein was detected in the injected muscle, ipsilateral sciatic nerve, and dorsal root ganglia (L4 and L5). In the ipsilateral tibia, luciferase expression was also detected, in accordance with the observed enzymatic activity, particularly in the interior of the metaphysis (Fig. 2F). High-magnification cross-sectional images showed that the luciferase protein was expressed in a tube-like structure known as a Haversian or Volkmann's canal in the cortical bone (Figs. 2G and H). In the cortical bone, Haversian canals run longitudinally down the center of the osteon, and Volkmann's canals run perpendicularly to interconnect with the Haversian canals. Both Haversian and Volkmann's canals contain an arteriole, a venule, a lymph duct, and a nerve fiber [18]. From the section stained with the anti-CGRP antibody (Fig. 2G), we assumed that nerves in the interior of the tibia expressed luciferase protein, because CGRP is contained in peripheral sensory nerves and distributed in bone via sensory nerves. No immunoreactivity was detected in the control samples, i.e., the contralateral tibialis anterior muscle, sciatic nerve, dorsal root ganglia, and tibia (Figs. 2A–C and F).

Note that no infiltration of inflammatory mononuclear cells was detected in the cortical bone examined at any defined time point (data not shown), indicating a low immunogenicity of this nonviral vector.

### Axonal transport mediates gene transduction in the interior of bone

**Figure 3:**
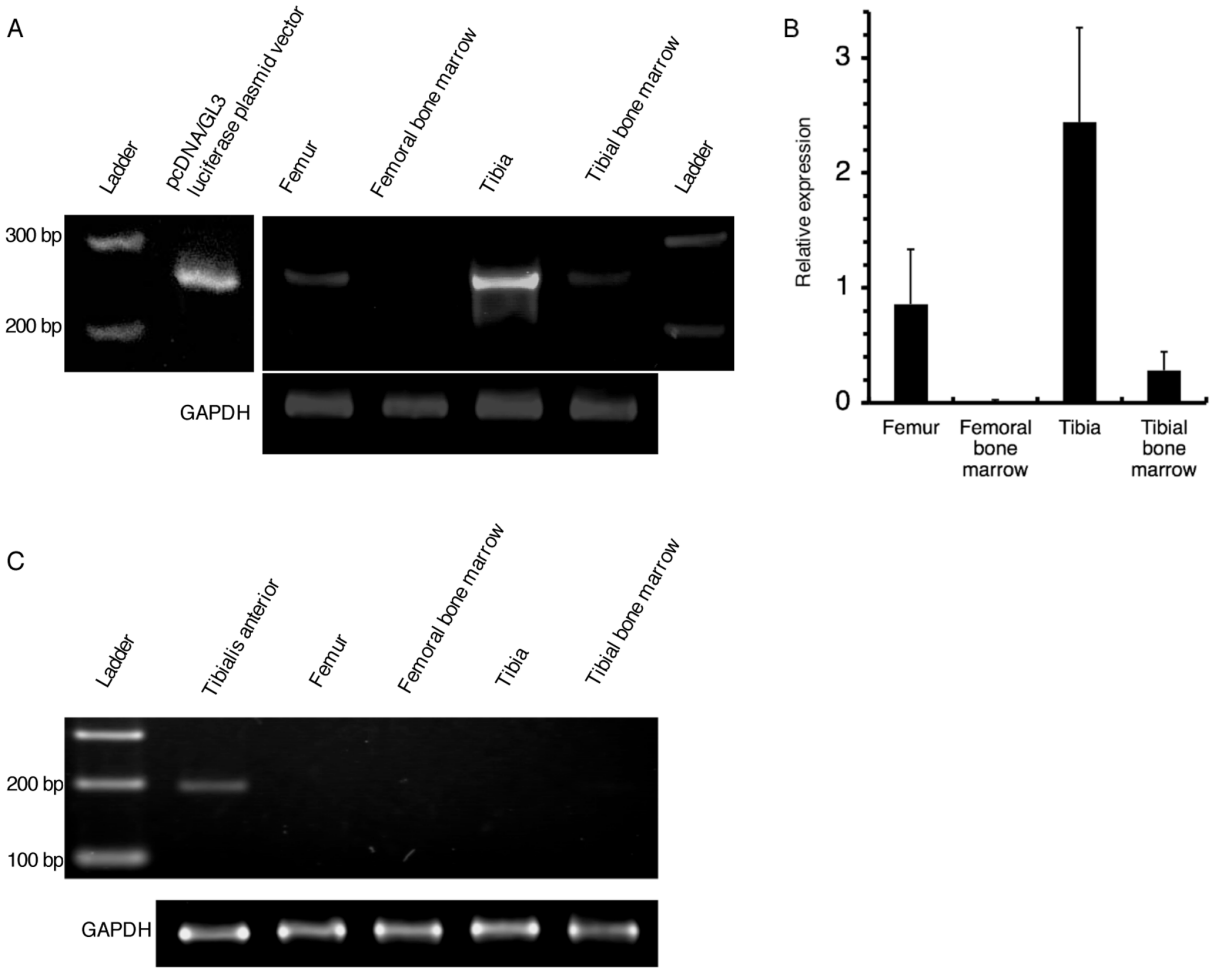
Expression of luciferase mRNA in the bone. (A) RT-PCR was performed with a primer specific for firefly luciferase (product length, 261 bp). Luciferase mRNA was detected in the rat RNA extracted from the femur as well as the tibia. (B) Using four rats, RT-PCR for luciferase mRNA was performed 4 times. Quantification was performed using Image J software (n = 4). Error bars: SEM. (C) To ensure that there was no contamination of tibialis anterior muscle in any of the bone samples, RT-PCR was performed using all the above samples and a primer specific for MHC IIb (product length, 197 bp). MHC IIb mRNA was not detected in the ipsilateral femora, ipsilateral tibiae, or bone marrows from ipsilateral femora and tibiae (n = 4).

The observation that nerves express the transduced luciferase gene lends support to the notion that axonal transport is crucial for gene delivery into bone. If so, we assumed that luciferase gene products would also be expressed in the ipsilateral femur and ipsilateral tibia. Luciferase mRNA was detected in the ipsilateral femur, albeit at a low level, by RT-PCR analysis using primers specific for firefly luciferase (Figs. 3A and B). However, we detected no luciferase proteins in the ipsilateral femur, possibly owing to the low expression level and threshold for the immunohistochemical detection (data not shown).

To ensure that there was no contamination of tibialis anterior muscle in any of the bone samples, RT-PCR was performed using all the above samples and a primer specific for MHC IIb, which is specifically expressed in fast-twitch muscle. MHC IIb mRNA was not detected in the ipsilateral femora, ipsilateral tibiae, or bone marrows from ipsilateral femora and tibiae (n = 4) (Figs. 3C).

**Figure 4:**
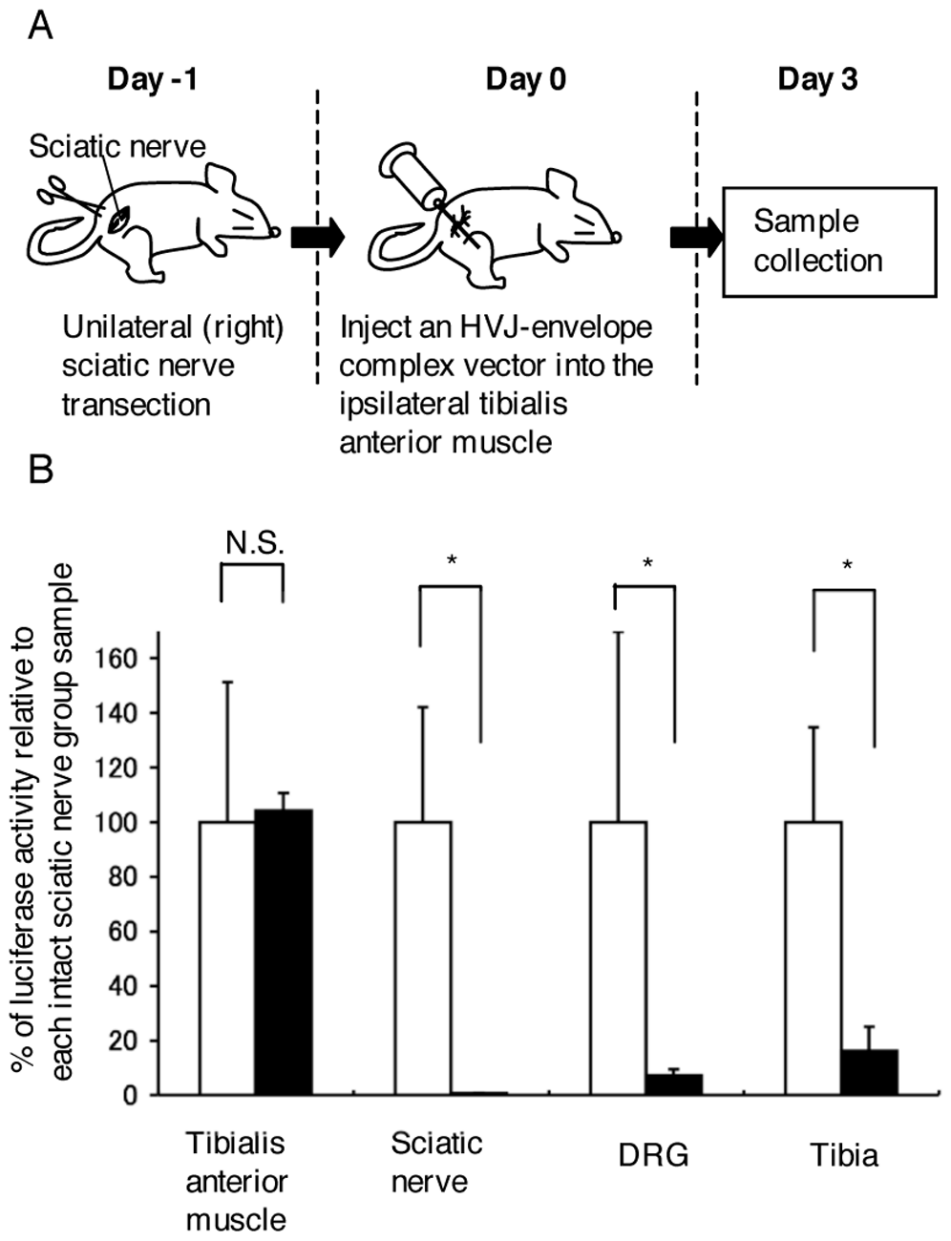
Sciatic nerve transection experiment. (A) Experimental design of sciatic nerve transection. The unilateral (right) sciatic nerves were transected 1 day before injecting an HVJ-envelope complex vector. HVJ-envelope-DNA complex vector injection was performed in the injected tibialis anterior muscle. Samples were harvested 3 days after the administration of a luciferase/HVJ-E vector. (B) Comparison of luciferase activities between the intact sciatic nerve group samples (white bars) and the sciatic nerve transection group samples (black bars) on day 3 after gene transduction (n = 7). Error bars: SEM. *p<0.05.

To further clarify the role of axonal transport in gene delivery into bone, we next examined the effect of sciatic nerve transection. Sciatic nerves were cut prior to the injection of a vector and samples were harvested on day 3 after the administration (Fig. 4A). We found that sciatic nerve transection makes the gene expression ineffectual in the tibia as well as in the sciatic nerve and dorsal root ganglia (L4 and L5), while the gene expression in the tibialis anterior muscle was not affected (Fig. 4B). It is tempting to speculate that the luciferase gene (or its gene products) may be conveyed into bones mainly via axonal transport, although we could not exclude the minor contribution via the blood or the lymphatics. Furthermore, we should take the lack of muscle contraction into account, because tibialis anterior muscles were not able to contract actively after sciatic nerve transection.

## Discussion

Local gene delivery into bone is problematic owing to the limited accessibility of the interior of bone, and its trials have remained preclinical for a long time [1], [13]. In this study, we demonstrated that efficient gene expression within bone is simply achieved by an intramuscular vector injection with a minimal immune response allowing repeated administrations. Although the detailed mechanism of this gene transduction remains uncertain, we suggest that gene expression within bones may be predominantly mediated by axonal transport.

**Figure 5:**
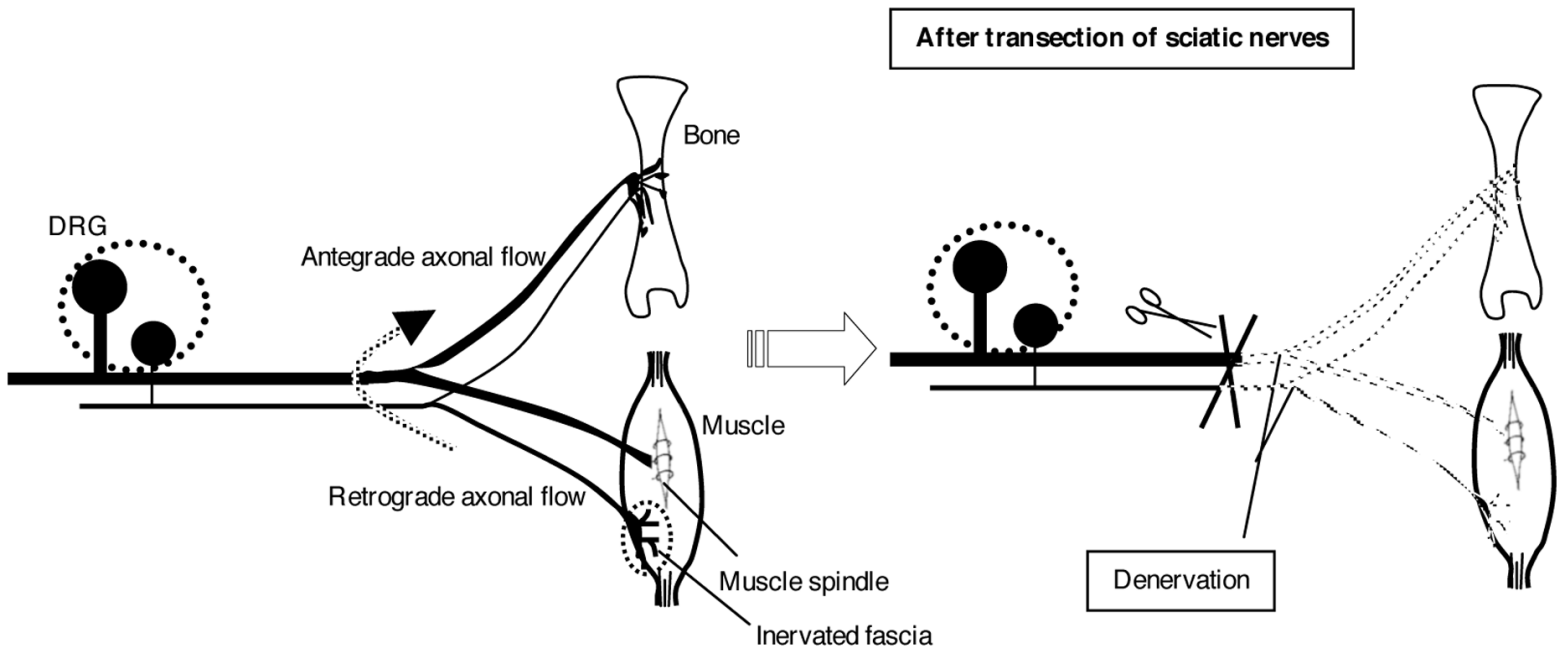
Possible mechanisms of gene transduction via axonal transport. If axons innervating both bone and muscle exist, gene expression could occur within the bone via axonal transport. The plasmid vector or its gene product could be transported retrogradely to the point of division of an axon. Next, we speculate two pathways of the plasmid vector or its gene product. In one pathway, a plasmid vector might be transported to DRG by fast retrograde axonal transport, and transcribed in DRG. Then, its gene product would be conveyed to peripheral sites by fast antegrade axonal transport. In the other pathway, a plasmid vector is conveyed to the other branch of the axon at its division point, and then reaches the bone. The right column shows the denervation of sciatic nerves after transection. Scissors show cut points. In this situation, no antegrade or retrograde axonal flow exists.

Many studies have reported evidence for the existence of dichotomizing axons of primary sensory neurons [26], [27], although there has been no report detailing how many sensory neurons project to both tibia and tibialis anterior muscle. If dorsal root ganglion neurons with dichotomizing axons innervating both bone and muscle exist, we could speculate two mechanisms of gene transduction in the interior of bone (Fig. 5). Large numbers of sensory nerves innervate not only fasciae but also muscle spindles [28], [29], and it has been reported that nonviral vectors may assist the retrograde axonal gene transfer and the reporter gene product may also be synthesized in DRG [30], [31]. On the basis of the above reports and our results that the levels of luciferase activity peaked on day 3 after gene transfection, we speculate first that the vector may be transported to DRG and back to peripheral sites by fast axonal transport. However, the ipsilateral tibia showed a high luciferase activity on day 1(Fig. 1B). Therefore, it is unlikely that the vector is transported to DRG and back to peripheral sites in only 1 day, even if conveyed by fast axonal transport, because fast retrograde axonal transport has been observed at rates of 100–410 mm in 1 day [32]–[34]. Next, we consider it possible that the plasmid vector or its gene product is transported to sciatic nerves via retrograde axonal flow from the tibialis anterior muscle, conveyed to the other branch of the axon at its division point, and then reaches the bone via antegrade axonal flow. However, detailed characterization of such pathways is required for better understanding of axonal-transport-mediated gene transduction. Meanwhile, when we introduce an exogenous gene into peripheral nerves to treat nerve disorders, we should consider that gene expression might also occur within bone via axonal transport.

In conclusion, we have demonstrated that the injection of an HVJ-envelope-DNA complex into an innervated muscle can achieve efficient gene expression in the interior of bone, and that sustained gene expression can be obtained by repeated gene transfers. Although we carried out only a short-term analysis, our findings may provide new insights into the development of a possible therapeutic alternative for hard-tissue disorders.

## References

[1] Evans CH, Ghivizzani SC, Robbins PD (2009). Orthopedic gene therapy in 2008.. Mol Ther.

[2] Partridge KA, Oreffo RO (2004). Gene delivery in bone tissue engineering: progress and prospects using viral and nonviral strategies.. Tissue Eng.

[3] Mason JM, Grande DA, Barcia M, Grant R, Pergolizzi RG (1998). Expression of human bone morphogenic protein 7 in primary rabbit periosteal cells: potential utility in gene therapy for osteochondral repair.. Gene Ther.

[4] Gouze E, Pawliuk R, Gouze JN, Pilapil C, Fleet C (2003). Lentiviral-mediated gene delivery to synovium: potent intra-articular expression with amplification by inflammation.. Mol Ther.

[5] Lieberman JR, Daluiski A, Stevenson S, Wu L, McAllister P (1999). The effect of regional gene therapy with bone morphogenetic protein-2-producing bone-marrow cells on the repair of segmental femoral defects in rats.. J Bone Joint Surg Am.

[6] Krebsbach PH, Gu K, Franceschi RT, Rutherford RB (2000). Gene therapy-directed osteogenesis: BMP-7-transduced human fibroblasts form bone in vivo.. Hum Gene Ther.

[7] Alden TD, Beres EJ, Laurent JS, Engh JA, Das S (2000). The use of bone morphogenetic protein gene therapy in craniofacial bone repair.. J Craniofac Surg.

[8] Ashinoff RL, Cetrulo CL, Galiano RD, Dobryansky M, Bhatt KA (2004). Bone morphogenic protein-2 gene therapy for mandibular distraction osteogenesis.. Ann Plast Surg.

[9] Baltzer AW, Lattermann C, Whalen JD, Wooley P, Weiss K (2000). Genetic enhancement of fracture repair: healing of an experimental segmental defect by adenoviral transfer of the BMP-2 gene.. Gene Ther.

[10] Baltzer AW, Lattermann C, Whalen JD, Ghivizzani S, Wooley P (2000). Potential role of direct adenoviral gene transfer in enhancing fracture repair.. Clin Orthop Relat Res.

[11] Helm GA, Alden TD, Beres EJ, Hudson SB, Das S (2000). Use of bone morphogenetic protein-9 gene therapy to induce spinal arthrodesis in the rodent.. J Neurosurg.

[12] Evans CH, Robbins PD (1995). Possible orthopaedic applications of gene therapy.. J Bone Joint Surg Am.

[13] Wazen RM, Moffatt P, Zalzal SF, Daniel NG, Westerman KA (2006). Local gene transfer to calcified tissue cells using prolonged infusion of a lentiviral vector.. Gene Ther.

[14] Kostenuik PJ, Bolon B, Morony S, Daris M, Geng Z (2004). Gene therapy with human recombinant osteoprotegerin reverses established osteopenia in ovariectomized mice.. Bone.

[15] Bolon B, Carter C, Daris M, Morony S, Capparelli C (2001). Adenoviral delivery of osteoprotegerin ameliorates bone resorption in a mouse ovariectomy model of osteoporosis.. Mol Ther.

[16] Verma IM, Weitzman MD (2005). Gene therapy: twenty-first century medicine.. Annu Rev Biochem.

[17] Newman KD, Dunn PF, Owens JW, Schulick AH, Virmani R (1995). Adenovirus-mediated gene transfer into normal rabbit arteries results in prolonged vascular cell activation, inflammation, and neointimal hyperplasia.. J Clin Invest.

[18] Marieb EN, Mallat J, Schmid J (1997). Human Anatomy;. Rand McNally.

[19] Wittenberg RH, Peschke U, Botel U (1992). Heterotopic ossification after spinal cord injury. Epidemiology and risk factors.. J Bone Joint Surg Br.

[20] Mendelson L, Grosswasser Z, Najenson T, Sandbank U, Solzi P (1975). Periarticular new bone formation in patients suffering from severe head injuries.. Scand J Rehabil Med.

[21] Cundy TF, Edmonds ME, Watkins PJ (1985). Osteopenia and metatarsal fractures in diabetic neuropathy.. Diabet Med.

[22] Carpintero P, Logrono C, Carreto A, Carrascal A, Lluch C (1998). Progression of bone lesions in cured leprosy patients.. Acta Leprol.

[23] Kato N, Nakanishi K, Nemoto K, Morishita R, Kaneda Y (2003). Efficient gene transfer from innervated muscle into rat peripheral and central nervous systems using a non-viral haemagglutinating virus of Japan (HVJ)-liposome method.. J Neurochem.

[24] Bert AG, Burrows J, Osborne CS, Cockerill PN (2000). Generation of an improved luciferase reporter gene plasmid that employs a novel mechanism for high-copy replication.. Plasmid.

[25] Kaneda Y, Nakajima T, Nishikawa T, Yamamoto S, Ikegami H (2002). Hemagglutinating virus of Japan (HVJ) envelope vector as a versatile gene delivery system.. Mol Ther.

[26] Ohtori S, Takahashi K, Chiba T, Yamagata M, Sameda H (2003). Calcitonin gene-related peptide immunoreactive neurons with dichotomizing axons projecting to the lumbar muscle and knee in rats.. Eur Spine J.

[27] Sameda H, Takahashi Y, Takahashi K, Chiba T, Ohtori S (2001). Primary sensory neurons with dichotomizing axons projecting to the facet joint and the sciatic nerve in rats.. Spine (Phila Pa 1976).

[28] Hunt CC (1954). Relation of function to diameter in afferent fibers of muscle nerves.. J Gen Physiol.

[29] Sherrington CS (1894). On the Anatomical Constitution of Nerves of Skeletal Muscles; with Remarks on Recurrent Fibres in the Ventral Spinal Nerve-root.. J Physiol.

[30] Thakor D, Spigelman I, Tabata Y, Nishimura I (2007). Subcutaneous peripheral injection of cationized gelatin/DNA polyplexes as a platform for non-viral gene transfer to sensory neurons.. Mol Ther.

[31] Wang S, Ma N, Gao SJ, Yu H, Leong KW (2001). Transgene expression in the brain stem effected by intramuscular injection of polyethylenimine/DNA complexes.. Mol Ther.

[32] Lundborg G (2002). Nerve injury and repair..

[33] Tomishima MJ, Smith GA, Enquist LW (2001). Sorting and transport of alpha herpesviruses in axons.. Traffic.

[34] Brown A (2003). Axonal transport of membranous and nonmembranous cargoes: a unified perspective.. J Cell Biol.

